# Hypertension Knowledge-Level Scale (HK-LS): A Study on Development, Validity and Reliability

**DOI:** 10.3390/ijerph9031018

**Published:** 2012-03-22

**Authors:** Sultan Baliz Erkoc, Burhanettin Isikli, Selma Metintas, Cemalettin Kalyoncu

**Affiliations:** Public Health Department, Medical Faculty, Eskisehir Osmangazi University,Meselik-Eskisehir 26480, Turkey; Email: burhan@ogu.edu.tr (B.I.); selmamet@ogu.edu.tr (S.M.); kalyoncu@ogu.edu.tr (C.K.)

**Keywords:** hypertension, knowledge, scale, validity, reliability

## Abstract

This study was conducted to develop a scale to measure knowledge about hypertension among Turkish adults. The Hypertension Knowledge-Level Scale (HK-LS) was generated based on content, face, and construct validity, internal consistency, test re-test reliability, and discriminative validity procedures. The final scale had 22 items with six sub-dimensions. The scale was applied to 457 individuals aged ≥18 years, and 414 of them were re-evaluated for test-retest reliability. The six sub-dimensions encompassed 60.3% of the total variance. Cronbach alpha coefficients were 0.82 for the entire scale and 0.92, 0.59, 0.67, 0.77, 0.72, and 0.76 for the sub-dimensions of definition, medical treatment, drug compliance, lifestyle, diet, and complications, respectively. The scale ensured internal consistency in reliability and construct validity, as well as stability over time. Significant relationships were found between knowledge score and age, gender, educational level, and history of hypertension of the participants. No correlation was found between knowledge score and working at an income-generating job. The present scale, developed to measure the knowledge level of hypertension among Turkish adults, was found to be valid and reliable.

## 1. Introduction

According to the World Health Organization (WHO), in 2008, an estimated 36 million of the 57 million worldwide deaths were due to non-communicable diseases (NCD). These diseases included primarily cardiovascular diseases, cancers, chronic respiratory diseases and diabetes, including approximately 9 million deaths before the age of 60, with nearly 80% of these deaths occurring in developing countries [1]. Hypertension had a prevalence of 26.4% in the worldwide adult population in 2000 (26.6% in men and 26.1% in women). The total number of hypertensive adults was 972 million: 333 million were in more economically developed countries, and 639 million were in less economically developed countries [2]. Furthermore, hypertension is one of the leading causes of premature death worldwide, accounting for 7.6 million deaths in 2001 [3]. The number of adults with hypertension in 2025 was predicted to increase by 60% to a total of 1.56 billion adults [2]. Hypertension is the most common chronic disease with sudden onset, and it is called the “silent killer” because it progressively and permanently damages organs. Hypertension causes several heart, brain and kidney diseases, resulting in severe and life-threatening complications, as well as death [4].

Non-communicable diseases including hypertension are among the leading causes of preventable morbidity and related disability. The monitoring of hypertension and other NCDs consists of country-level surveillance and monitoring systems, including surveys that are integrated into existing national health information systems and that include monitoring exposure to risk factors, outcomes, social and economic determinants of health, and health system responses [1].

Hypertension is common in Turkey, as it is worldwide [5]. The Turkish Hypertension Prevalence Study (the PatenT study) found that the prevalence of hypertension was 31.8% (27.5% in men and 36.1% in women) in adults aged 18 years and over. Only 40.7% were aware of their disease, and 31.1% were on antihypertensive treatment in 2003. Controlled blood pressure was observed in 8% of all hypertensive patients and in 20.7% of patients who were aware of their high blood pressure and receiving antihypertensive treatment [6]. According to data from the 2004 National Burden of Disease Study, controlling high blood pressure in adults aged 30 years and over would prevent deaths in 20.4% of men and 30.8% of women [7].

The management and control of hypertension is possible with a combination of medication and strict lifestyle changes. Control programs are required to manage hypertension in a social dimension. However, it is well known that the control of hypertension is inadequate in Turkey and in many other countries [8]. The main reasons for this inadequate control of blood pressure include demographic characteristics, health beliefs and the presence of other chronic diseases. Other reasons include lack of hypertension awareness and lack of knowledge about high blood pressure [4,9]. While it is difficult or impossible to change demographic and personal characteristics, cultural norms and socioeconomic status, increasing knowledge through educational interventions on treatment can positively influence patients’ beliefs about medicines [10]. Because hypertension may occur for many people at some point in their lives, safe and potentially effective preventive measures should be more widely established [11].

Although instruments have been used in studies conducted on various populations to determine the knowledge level on hypertension and risk factors, there are no such valid and reliable instruments for the Turkish population [12,13]. Patients’ knowledge level can be increased by educational interventions. The determination of the current level of knowledge would shed light on future educational intervention studies. The present study was conducted to develop an instrument for measuring the knowledge level of Turkish adults concerning hypertension and to establish the instrument’s validity and reliability.

## 2. Methods

### 2.1. Establish Face and Content Validity of the Hypertension Knowledge-Level Scale (HK-LS)

Recent data from the literature, the Turkish Society of Cardiology treatment and follow-up guidelines, and treatment guidelines from several international hypertension associations were reviewed for the development of the scale [14,15,16,17,18].

During the development of the Hypertension Knowledge Level Scale (HK-LS), a 52–item scale was prepared by researchers [9,10,11,14,15,16,17,18,19,20,21]. Each item was a full sentence that was either correct or incorrect. The items questioned the definition, etiology, medical treatment and complications of hypertension, as well as the attitudes and behaviors about drug compliance, diet, and lifestyle. Each item was prepared as part of a standard answer (Correct, Incorrect or Don’t Know). In an effort to assess the content validity of the scale to identify whether items were or were not representative of the knowledge level of hypertension, the opinions of nine experts (one sociologist, three epidemiologists, two cardiologists, one primary care physician, one pharmacologist, and one internal medicine specialist) were requested via an assessment form. The experts were asked to grade each item as “essential,” “useful but inadequate” or “unnecessary”. The expert opinions were evaluated, and 12 items were excluded from the scale. All items were evaluated in terms of clarity and expression by considering the expert opinions, and relevant changes were made.

A convenience sample of ten individuals who did not have medical or research backgrounds were also asked to provide feedback on the questionnaire in terms of its language and clarity. This sample included adult professionals, such as hospital personnel and schoolteachers.

### 2.2. Study Group and Procedure

Approval by the local committee (certification number: 2011/260) and verbal consent from the participants were received prior to participants’ enrollment in the study.

The study was conducted in Alpu County (Eskisehir, Turkey) between July 2011 and August 2011. The study population was composed of all adults over 18 years of age living in Alpu. The sample size was calculated at 400 individuals by considering the statement “sample size should be 5 to 10 times the number of items in the study scale” [22]. However, we assumed that individuals may be lost during test-retest, and therefore the sample size was increased by 15%, for a total of 460 individuals included in the study. All adults over the age of 18 living in three of the four lottery-selected towns (Fatih, Kemal Pasa and Yunus Emre) of Alpu were visited individually. All enrolled subjects were visited in their homes by Erkoc SB, one of the study authors, and by intern doctors who were trained on the questionnaire, which was completed during face-to-face interviews.

Individuals with cognitive dysfunction preventing them from understanding the questions or giving clear answers, individual visitors to the study area, and individuals who did not agree to participate in the study were excluded.

The HK-LS scale was applied to all participants, and demographic characteristics (age, gender, education level, and work at income-generating job and personal and/or family history of hypertension were examined. Information was obtained during a 15 to 30 min face-to-face conversation.

### 2.3. Statistical Analysis

SPSS version 13.0 for Windows (SPSS Inc., Chicago, IL, USA) was used for the data analysis. The demographic characteristics of the study group were reported by using descriptive statistics (frequencies, proportions, and means). The mean scores were compared by *t*-tests and one-way ANOVA.

### 2.4. Evaluation of the Hypertension Knowledge-Level Scale (HK-LS) Factor Analysis

In order to determine the qualifications measured by the scale and examine the meaning of the total scores, construct validity was assessed by factor analysis, specifically principal component analysis. Factor analysis adequacy was demonstrated by applying the Kaiser-Meyer-Olkin (KMO) test to the scale. Because the KMO result was >0.50, factor analysis was performed [23].

Single-item sub-dimension items and items with a factor loading of <0.49 (total: 13 items) in the factor analysis were excluded. Among the factor rotation methods, Equamax Rotation Method was selected. According to factor loading assessed by factor analysis, items pertained to a sub-dimension according to their maximum factor weight. Eight sub-dimensions were identified by factor analysis. 

#### 2.4.1. Internal Consistency

The Cronbach alpha coefficient was calculated for the reliability analysis of each sub-dimension. Items that had an item-total correlation of <0.40 were discarded from the measure. For each factor, a Cronbach coefficient alpha value of >0.59 and <0.95 was considered acceptable. Ultimately, 5 items in 2 sub-dimensions were also excluded from the scale. Correlation analysis was used to assess internal consistency reliability. The correlation coefficient must not be negative or below 0.20 [23]. 

#### 2.4.2. Test-Retest Reliability

The stability of the instrument over time was tested by the test-retest reliability method. Two weeks after the initial application, the scale was applied to 414 of 457 individuals (90.6%) for test-retest reliability. Spearman’s rank correlation coefficient was used to measure the level of agreement between responses at test and re-test.

#### 2.4.3. Discriminative Validity

Discriminative validity of the scale compares the group scores [24]. Knowledge levels are believed to be greater in individuals with personal and/or family histories of hypertension; therefore, the total scores of the study group were compared by *t*-test.

Subjects’ total scores on each item were put in order from lowest to highest for the assessment of internal criterion validity. The difference between the total scores of the lower 25% and the upper 25% of the distribution was analyzed by *t*-test (Student’s *t*-test). 

### 2.5. Scoring

The final scale had 22 items with six sub-dimensions. The expression was incorrect for 9 items. Each correct answer was worth 1 point. Incorrect statements were encoded inversely to the other items. The maximum score was 22 for the entire scale, 2 for “definition”, 4 for “medical treatment”, 4 for “drug compliance”, 5 for “lifestyle”, 2 for “diet”, and 5 for “complications” sub-dimensions. The minimum score was zero for the entire scale and for all sub-dimensions. 

## 3. Results

### 3.1. Study Group

The mean age of the 457 individuals was 44.56 ± 15.15 years (range, 18–82 years). Of these, 31.3% were male and 68.7% were female. A personal history of hypertension was observed in 23.2% of the participants, a family history in 34.8% and a personal and/or family history in 47.0%. The distribution of the study group according to various demographic and medical characteristics is shown in Table 1. 

**Table 1:** Distribution of the study group according to various demographic and medical characteristics.

Variables	Number ( *n* = 457)	Percentage (%)
Age group (years)		
18–29	83	18.2
30–39	102	22.3
40–49	105	23.0
50–59	80	17.5
60+	87	19.0
Gender		
Male	143	31.3
Female	314	68.7
Education level		
No formal education	57	12.5
1–8 years	253	55.3
9 years+	147	32.2
Work at income generating job		
No	289	63.2
Yes	168	36.8
Personal history of HT		
Absent	351	76.8
Present	106	23.2
Family history of HT		
Absent	298	65.2
Present	159	34.8

HT: Hypertension.

### 3.2. Evaluation of the Hypertension Knowledge-Level Scale (HK-LS)

#### 3.2.1. Factor Analysis

The Kaiser-Meyer-Olkin measure of sampling adequacy was 0.78. Bartlett’s test of sphericity was significant (*x*^2^ = 3300.796, df = 231, *P* < 0.001). Six sub-dimensions encompassed 60.3% of the total variance. The results of the factor analysis for HK-LS are shown in Table 2.

**Table 2:** Results of the factor analysis for HK-LS**.**

Sub-dimensions Item number	Item	Factor loading	Variation explained (%)
Definition			
1	Increased diastolic blood pressure also indicates increased blood pressure.	0.93	9.06
2	High diastolic or systolic blood pressure indicates increased blood pressure.	0.92	
Medical Treatment			
3	Drugs for increased blood pressure must be taken everyday.	0.72	
4	Individuals with increased blood pressure must take their medication only when they feel ill.	0.65	8.08
5	Individuals with increased blood pressure must take their medication throughout their life.	0.61	
6	Individuals with increased blood pressure must take their medication in a manner that makes them feel good.	0.49	
Drug Compliance			
7	If the medication for increased blood pressure can control blood pressure, there is no need to change lifestyles.	0.73	
8	Increased blood pressure is the result of aging, so treatment is unnecessary.	0.68	
9	If individuals with increased blood pressure change their lifestyles, there is no need for treatment.	0.66	10.58
10	Individuals with increased blood pressure can eat salty foods as long as they take their drugs regularly.	0.59	
Lifestyle			
11	Individuals with increased blood pressure can drink alcoholic beverages.	0.76	
12	Individuals with increased blood pressure must not smoke.	0.72	
13	Individuals with increased blood pressure must eat fruits and vegetables frequently.	0.65	
14	For individuals with increased blood pressure, the best cooking method is frying.	0.62	11.27
15	For individuals with increased blood pressure, the best cooking method is boiling or grilling.	0.61	
Diet			
16	The best type of meat for individuals with increased blood pressure is white meat.	0.83	9.16
17	The best type of meat for individuals with increased blood pressure is red meat.	0.69	
Complications			
18	Increased blood pressure can cause premature death if left untreated.	0.80	
19	Increased blood pressure can cause heart diseases, such as heart attack, if left untreated.	0.76	12.16
20	Increased blood pressure can cause strokes, if left untreated.	0.75	
21	Increased blood pressure can cause kidney failure, if left untreated.	0.62	
22	Increased blood pressure can cause visual disturbances, if left untreated.	0.62	

#### 3.2.2. Internal Consistency

The Cronbach alpha values were as follows: 0.82 for the entire scale, 0.92 for the definition, 0.59 for medical treatment, 0.67 for drug compliance, 0.77 for lifestyle, 0.72 for diet, and 0.76 for complications sub-dimension in the initial test. The coefficients for the second application were 0.83, 0.92, 0.57, 0.65, 0.74, 0.79 and 0.80, respectively, in the second test.

The average of the scale after excluding the item ranged between 16.75 and 17.17, the variation of the scale after excluding the item ranged between 12.43 and 13.63, and the average alpha value of the scale after excluding the item ranged between 0.80 and 0.82. The corrected items-total score correlation coefficient was a minimum of 0.27 and a maximum of 0.50 (items 12 and 14, respectively), and there were 10 items with a corrected items-total score correlation coefficient >0.40.

#### 3.2.3. Test-Retest Reliability

**Figure 1:**
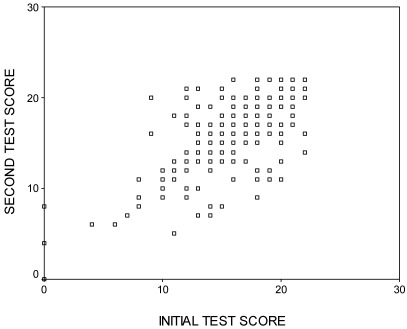
Scatter plot of the HK-LS test-retest scores.

Following the test-retest, a high positive correlation was observed between the total scores of the two applications using Spearman’s rank correlation analysis (*r* = 0.798, *P* < 0.001). A scatter plot of the HK-LS test-retest scores are shown in Figure 1. 

#### 3.2.4. Discriminative Validity

The total scale score was significantly higher in individuals with histories of hypertension compared to those without (18.08 ± 3.41 *vs*. 17.37 ± 4.04) (*t* = 2.022; *P* = 0.044). The scores for the medical treatment sub-dimension (*t* = 2.593; *P* = 0.010) and complications sub-dimension (*t* = 2.7750; *P* = 0.006) were significantly higher among individuals with histories of hypertension.

In the internal criterion validity assessment, the scores taken from 22 items are listed from lowest to highest. The total score for subjects in the lower 25% group (21.54 ± 0.51; *n* = 114) was higher than the total score for subjects in the top 25% group (12.53 ± 3.24; *n* = 114) (*t* = 29.26; *P* = 0.000). In other words, the scores for subjects placed at the bottom of the list (lower 25%) were higher than the scores for subjects placed at the top of the list (top 25%). 

### 3.3. Evaluation of the HK-LS Results of the Study Group

The distribution of sub-dimensions and total HK-LS scale according to various demographic characteristics of the study group are shown in Table 3.

**Table 3:** The distribution of sub-dimensions and total HK-LS scale according to various demographic characteristics of the study group.

Variables	Sub-dimension 1	Sub-dimension 2	Sub-dimension 3	Sub-dimension 4	Sub-dimension 5	Sub-dimension 6	Total Scale
	(Mean ± SD)	(Mean ± SD)	(Mean ± SD)	(Mean ± SD)	(Mean ± SD)	(Mean ± SD)	(Mean ± SD)
TOTAL	1.09 ± 0.96	3.08 ± 1.09	3.14 ± 1.15	4.55 ± 1.02	1.73 ± 0.60	4.13 ± 1.31	17.71 ± 3.77
Age group (y)							
18–29	1.33 ± 0.95	3.04 ± 1.13	3.35 ± 1.12	4.49 ± 1.21	1.68 ± 0.72	4.11 ± 1.28	17.76 ± 4.04
30–39	1.20 ± 0.93	3.23 ± 1.10	3.20 ± 1.08	4.61 ± 0.90	1.74 ± 0.58	4.09 ± 1.21	18.05 ± 3.44
40–49	1.03 ± 0.98	3.17 ± 0.98	3.18 ± 1.09	4.56 ± 1.06	1.82 ± 0.46	4.37 ± 1.19	18.13 ± 3.28
50–59	1.10 ± 0.96	3.10 ± 1.06	3.15 ± 1.07	4.69 ± 0.63	1.79 ± 0.52	4.34 ± 1.12	18.16 ± 3.20
60+	0.99 ± 0.98	2.81 ± 1.10	2.80 ± 1.33 *	4.40 ± 1.17	1.62 ± 0.72	3.70 ± 1.63 *	16.33 ± 4.57 *
Statistical analysis	*P* = 0.591	*P* = 0.100	***P* = 0.025**	*P* = 0.405	*P* = 0.122	***P* = 0.004**	***P* = 0.004**
Gender							
Male	1.09 ± 0.96	2.90 ± 1.25	3.01 ± 1.27	4.53 ± 1.07	1.73 ± 0.59	4.03 ± 1.41	17.30 ± 4.26
Female	1.09 ± 0.96	3.16 ± 1.00 *	3.19 ± 1.08	4.56 ± 0.99	1.72 ± 0.61	4.16 ± 1.26	17.89 ± 3.52
Statistical analysis	*P* = 0.986	***P* = 0.025**	*P* = 0.150	*P* = 0.801	*P* = 0.853	*P* = 0.312	*P* = 0.148
Education level							
No formal ed.	0.82 ± 0.97	2.82 ± 1.09	2.56 ± 1.35 *	1.53 ± 0.76 *	1.53 ± 0.76 *	3.70 ± 1.69 *	15.56 ± 4.61 *
1–8 years	1.05 ± 0.96	3.06 ± 1.11	3.08 ± 1.13 *	1.76 ± 0.57	1.76 ± 0.57	4.16 ± 1.29	17.75 ± 3.55
9 years+	1.27 ± 0.93 *	3.20 ± 1.06	3.45 ± 0.99 *	1.74 ± 0.59	1.74 ± 0.59	4.24 ± 1.15	18.46 ± 3.48
Statistical analysis	***P* = 0.007**	*P* = 0.079	***P*=0.000**	***P* = 0.002**	***P* = 0.026**	***P* = 0.027**	***P* = 0.000**
Work at income generating job							
No	1.06 ± 0.97	3.14 ± 1.02	3.16 ± 1.10	4.56 ± 1.00	1.72 ± 0.61	4.13 ± 1.30	17.76 ± 3.56
Yes	1.15 ± 0.95	2.98 ± 1.21	3.10 ± 1.23	4.53 ± 1.04	1.73 ± 0.60	4.13 ± 1.34	17.61 ± 4.11
Statistical analysis	*P* = 0.317	*P* = 0.145	*P* = 0.567	*P* = 0.755	*P* = 0.879	*P* = 0.981	*P* = 0.677

* = The group difference..

Mean scores taken from drug compliance, complication sub-dimensions and total scale were lower in individuals aged sixty years or older (*P* < 0.05). The medical treatment sub-dimension mean scores were higher in women (*P* < 0.05). Lifestyle, diet, complication sub-dimensions, and total scale mean scores were lower in individuals who had no formal education. Individuals with nine or more years of formal education had higher mean scores for the definition sub-dimension (*P* < 0.05). For the drug compliance sub-dimension, mean scores increased significantly as education level increased (*P* < 0.05). No differences were observed between individuals who had income-generating jobs and those who did not in terms of mean scores for all sub-dimensions and total scale (*P* > 0.05).

## 4. Conclusions

Hypertension progressively and permanently damages target organs, leading to life-threatening complications and death [4]. Chronic diseases, such as hypertension, necessitate lifelong drug intake and changes in lifestyle. A lack of knowledge about hypertension negatively influences patients’ awareness and behaviors, and is a major obstacle in controlling the disease [25]. Educational interventions are necessary to control hypertension [20]. The present study was conducted to develop a scale that accurately reflects culturally consistent social norms, standards and viewpoints in an attempt to determine individual knowledge levels.

Validity is the extent to which an instrument measures the target issue without mistaking it with another issue. Reliability is a prerequisite for regarding a measurement as valid. Reliability is the extent to which an instrument gives consistent results in repeated measurements under similar conditions. Although reliability is a prerequisite for validity, it is not sufficient on its own for validity. Scales that are reliable may not necessarily be valid [26].

The factor analysis method is used to group interdependent variables into a specific cluster [23]. Twenty-two items in six sub-dimensions were determined following the factor and reliability analysis of HK-LS.

The Cronbach alpha coefficient, which represents internal consistency reliability, should be higher than 0.70 [23]. Cronbach alpha coefficients for HK-LS were 0.82 for the whole scale and higher than 0.70 for all sub-dimensions, except for two (medical treatment and drug compliance), implying that the scale has considerable reliability. 

Although Cronbach alpha is an appropriate reliability coefficient for one-dimensional scales, it is advisable to use either test-retest or parallel-forms reliability methods in addition to alpha coefficients for multi-dimensional scales based on every single item [23].

Corrected item-total score correlation coefficients were calculated to estimate the contribution of items to the conceptual construct and whether those items can better measure a feature or not. Items with a corrected item-total score correlation coefficient of >0.40 are regarded as highly discriminative, those between 0.21 and 0.40 are regarded as somewhat discriminative, and those <0.20 are poorly discriminative [23]. The minimum corrected item-total score correlation coefficient of the items was 0.27, and there were 10 items with a corrected item-total score correlation coefficient of >0.40.

Scale stability over time, *i.e.*, the extent to which repeated applications of the instrument achieve consistent results, was assessed by the test-retest reliability method [23]. Test-retest is the application of an instrument twice to the same subjects under the same conditions, with a time interval that is long enough to prevent relevant recalls and that is short enough to disallow considerable changes in the construct being measured [26].

Statistically significant results for the test-retest reliability assessment of HK-LS with a 2-week time interval and a correlation coefficient of >70 indicate that the scale is stable over time (*r* = 0.798; *P* < 0.001). HK-LS test-retest reliability of the test results shows a strong positive correlation, indicating good stability.

Concurrent validity refers to the degree to which a newly developed scale correlates with another (equivalent) instrument that measures the same conceptual construct [23]. A major limitation of the present study was the lack of a valid and reliable equivalent scale for Turkish populations for the assessment of concurrent validity.

The validity analysis determined that HK-LS is appropriate for measuring hypertension knowledge levels. In addition, the reliability analysis determined that HK-LS can be used without measurement error, collects data accurately and is a repeatable scale [23].

The discriminative validity of a scale can also be assessed by comparing the scores of given groups [24]. The total scale scores of patients with a history of hypertension were significantly higher than those without (*t* = 2.022; *P* = 0.044).

In our study, significant relationships were observed between knowledge score and age, gender, educational level and history of hypertension, while no correlation was observed between knowledge score and having an income-generating job. Sabouhi *et al*. reported that there were significant relationships between knowledge score, age and education level, while there was no relationship in terms of gender. Martins *et al*. also reported a relationship between knowledge level and history of hypertension and education level [4,13].

HK-LS is the first scale that will be used in future studies for preventing hypertension, as well as for control programs and educational interventions to determine the hypertension knowledge level of Turkish adults. Although the present study was conducted on a representative group of the population, it has been shown that HK-LS has high validity and high reliability. However, further studies and developments are required to obtain further data on the validity and reliability of the scale.
